# Hybrid underwater endoscopic submucosal dissection : a safe and efficient training strategy for colorectal endoscopic submucosal dissection trainees

**DOI:** 10.1055/a-2900-8255

**Published:** 2026-08-18

**Authors:** Pier-Anne Gaulin, Remi Collin, Paul Carrier, Sophie Geyl, Jérémie Albouys, Romain Legros, Jeremie Jacques

**Affiliations:** 1 Hepatogastroenterology Department 36715 University Hospital Centre of LimogesLimogesAquitaine-Limousin-Poitou-CharentesFrance

Endoscopic submucosal dissection (ESD) training is predominantly concentrated in expert referral centers, where case selection favors advanced or technically challenging lesions, limiting hands-on opportunities for trainees. 1​ This is particularly relevant in Western practice, where colorectal ESD predominates and only a minority of lesions—estimated at up to 36%—are suitable for early training. 2​ These are typically small, non-fibrotic lesions in favorable locations. 3​ Therefore, safe and efficient strategies enabling progressive skill acquisition while maintaining procedural quality are essential. Hybrid ESD represents a valuable transitional approach, combining submucosal dissection with the option of completing resection using a snare when maneuverability or time constraints arise.

**Fig. 1:**
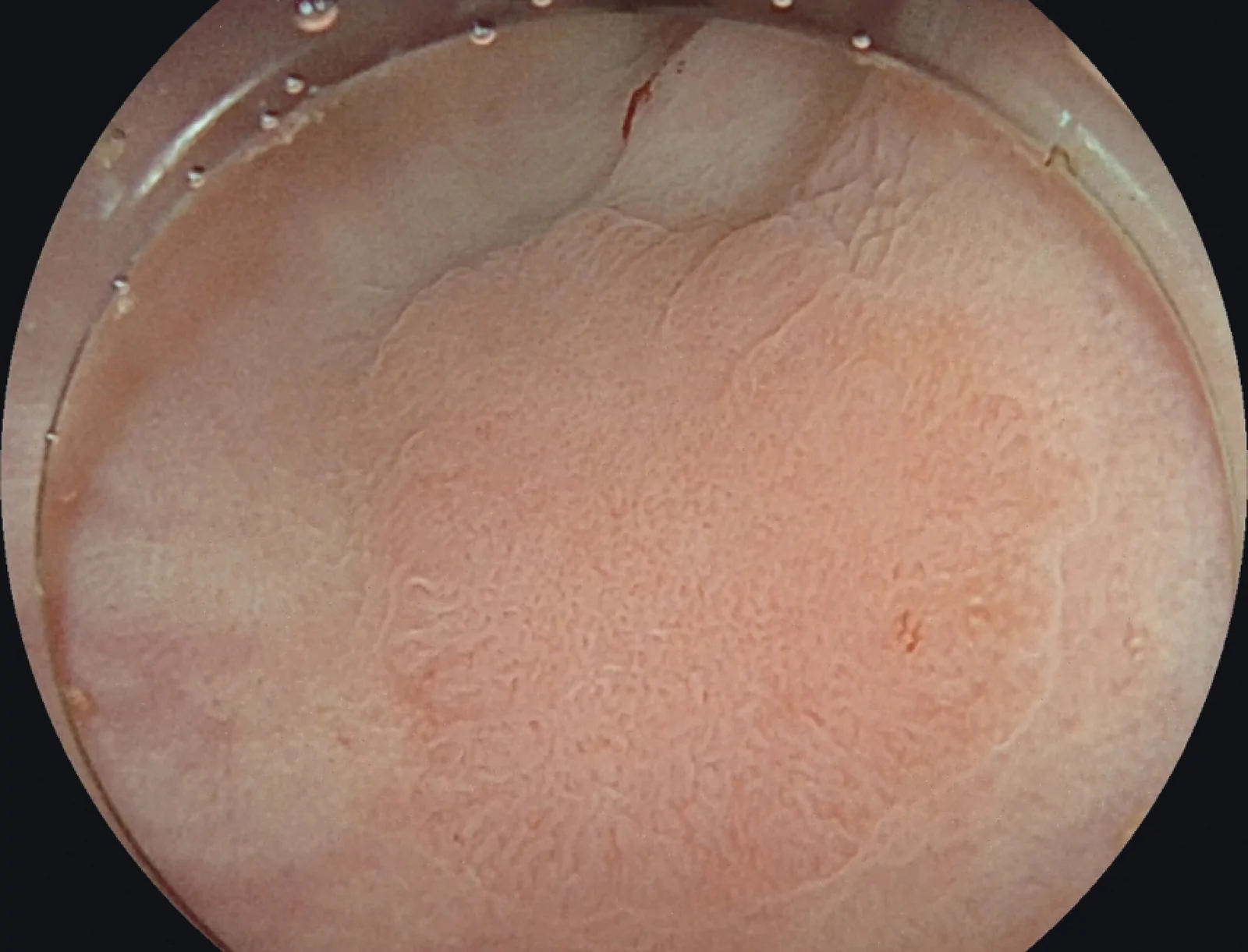
A 12-mm non-granular laterally spreading tumor with pseudo-depressed morphology in the transverse colon.

**Fig. 2:**
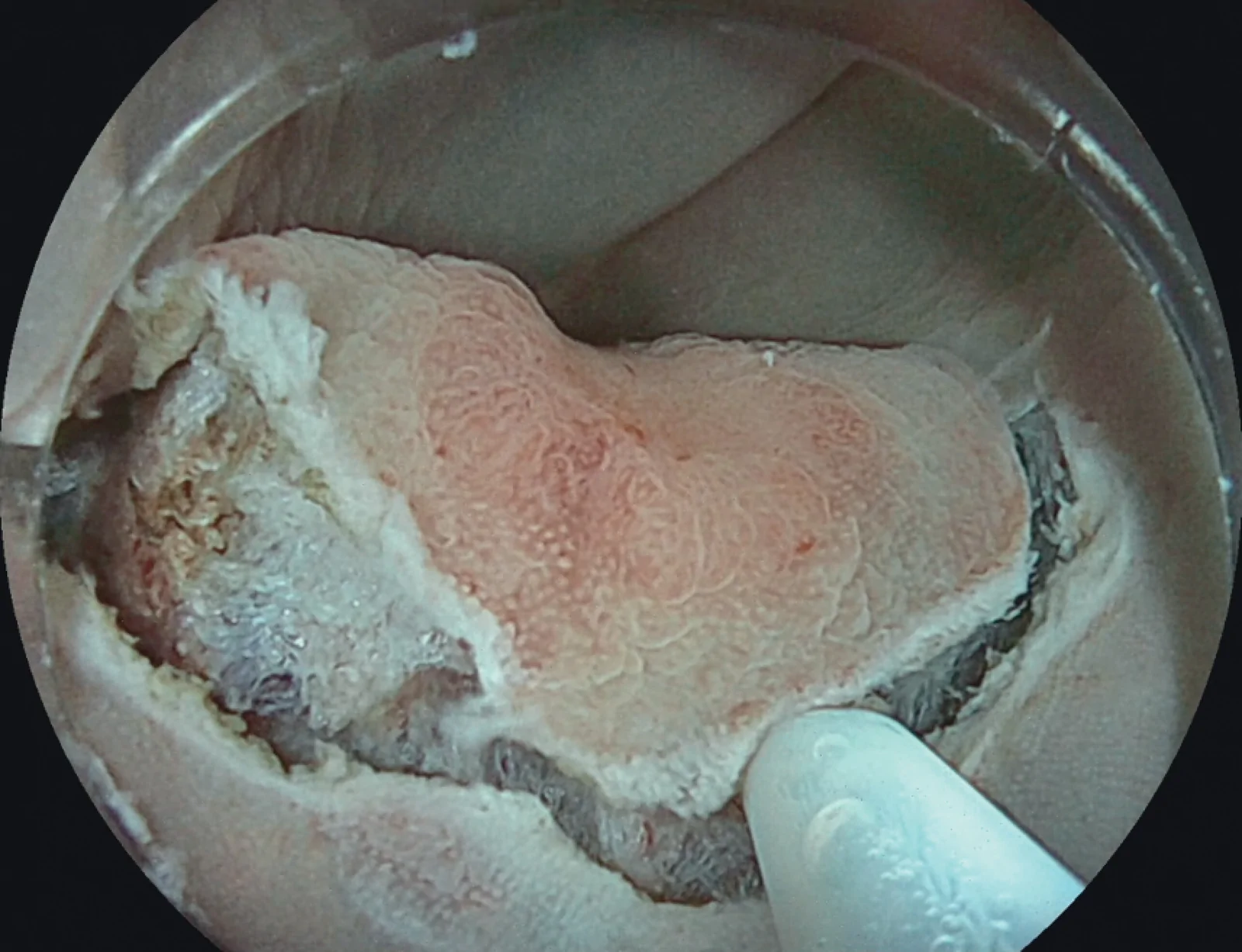
Submucosal dissection using the tip of a 15 mm hot snare.

**Fig. 3:**
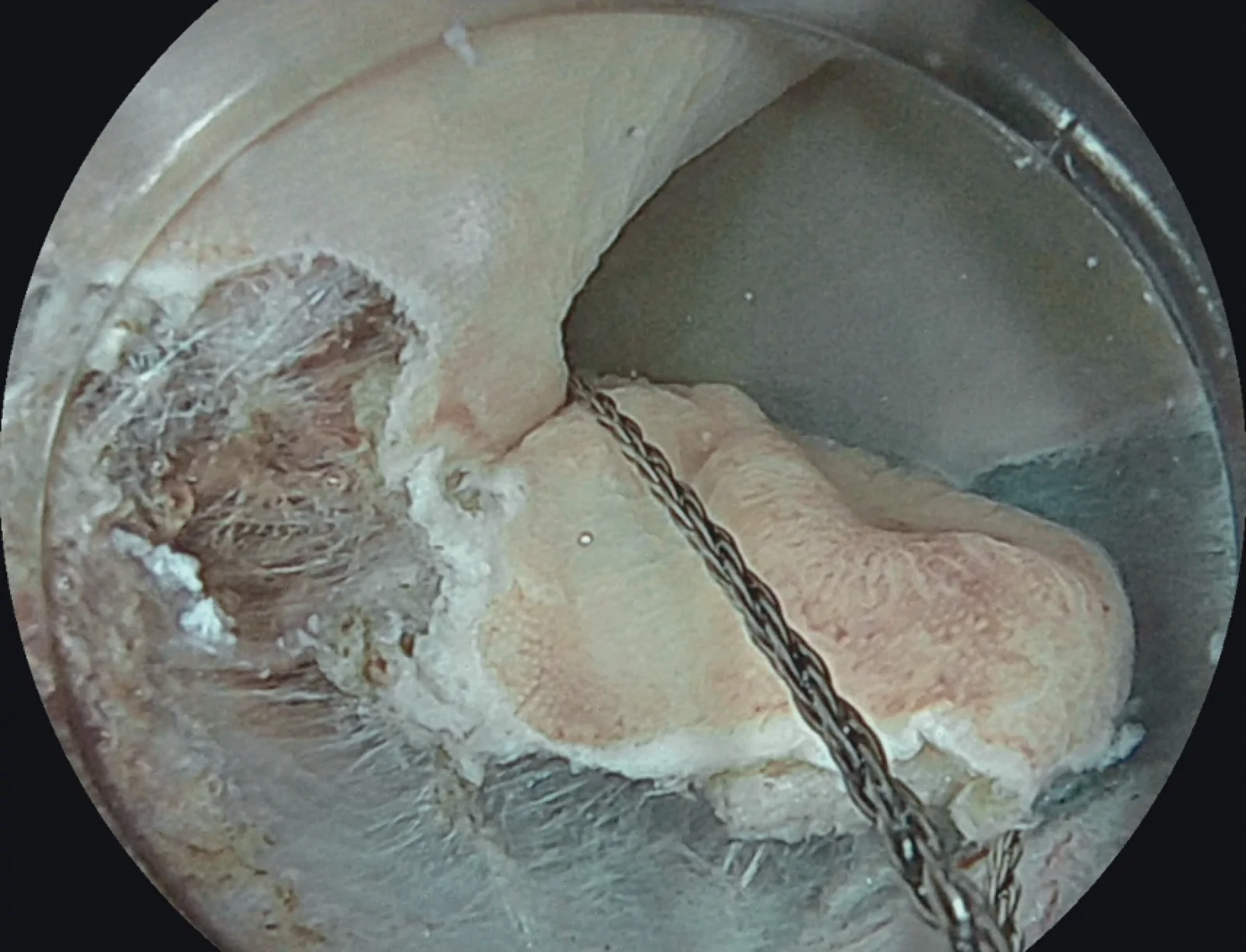
As maneuverability decreases, transition to underwater en bloc EMR after submucosal reinjection.

We report a 12-mm non-granular laterally spreading tumor with pseudo-depressed morphology in the transverse colon ( Fig. 1 ). After submucosal injection using a viscous solution, a circumferential mucosal incision was performed with the tip of a 15-mm hot snare, followed by trimming. Submucosal dissection was initiated using the snare tip in an underwater environment ( Fig. 2 ). Initially, given the adequate exposure of the submucosal plane thanks to the underwater technique, 4​ dissection was continued using the tip of the snare. Once maneuverability decreased during progression, the strategy was adapted to hybrid resection for safety reason, with reinjection followed by en bloc underwater EMR ( Fig. 3 ).

Inspection of the defect showed adequate hemostasis with limited superficial muscular exposure. En bloc resection was achieved in 17 minutes, yielding an 18-mm specimen with 14-mm low-grade dysplasia and R0 margins ( Video 1 ).

**Video 1** A 12-mm non-granular laterally spreading tumor with pseudo-depressed morphology in the transverse colon resected by hybrid underwater ESD.


This case highlights hybrid underwater ESD as a pragmatic and safe training approach. The extent of dissection completed before transitioning to snare resection may be tailored to submucosal plane exposure and endoscopic maneuverability. This allows the stepwise acquisition of key dissection skills while preserving procedural efficiency and resection quality.

Endoscopy_UCTN_Code_TTT_1AQ_2AD_3AD
